# Structural Descriptors and Antioxidant Activity Markers of 4-[4-(2-Aminoethoxy)benzyl]aniline

**DOI:** 10.3390/antiox15020256

**Published:** 2026-02-17

**Authors:** Dmitry A. Filimonov, Alexander B. Eresko, Nadezhda N. Trubnikova, Irina A. Kisilenko, Margarita A. Belotserkovskaya, Elena V. Raksha, Roman V. Ishchenko, Dorota M. Chudoba

**Affiliations:** 1 Federal State Budgetary Institution “V.K. Gusak Institute of Emergency and Reconstructive Surgery” of the Ministry of Health of the Russian Federation, Donetsk 283045, Russia; neuro.dnmu@gmail.com (D.A.F.);; 2 Frank Laboratory of Neutron Physics, Joint Institute for Nuclear Research, Dubna 141980, Russia

**Keywords:** thyronamines, ischemic stroke, oxidative stress markers, H_2_O_2_, DFT calculations, bond dissociation energy

## Abstract

The release of reactive oxygen species accompanying oxidative stress is one of the most significant damaging mechanisms during brain ischemia. Some aspects of the neuroprotective activity of the thyronamine T0AM synthetic analogue, 4-[4-(2-aminoethoxy)benzyl]aniline (ABA), were studied and discussed in two independent experiments using a model of acute cerebral ischemia. Antioxidant effects were evaluated in adult male and female Wistar rats (*Rattus norvegicus*), while neurological outcomes were assessed in adult male outbred white rats. Administration of the ABA in a rat brain hemisphere ischemia model was associated with significant changes in redox markers: malondialdehyde, glutathione peroxidase and superoxide dismutase levels in the ischemic hemisphere. Also, the introduction of ABA into the model of acute cerebral ischemia contributed to a reduction in neurological deficit compared to untreated controls. It was revealed that the considered thyronamine T0AM analogue could control redox status in acute brain ischemia. Mono protonated form of ABA (ABA-H^+^) is considered to be the main species at pH 6.9–7.2. Structural models of the neutral (ABA), protonated (ABA-H^+^) thyronamine and its solvate (DMSO…ABA-H^+^) were used in DFT calculations, followed by estimation of molecular and supramolecular level descriptors.

## 1. Introduction

Overproduction of cellular reactive oxygen and nitrogen species (ROS, RNS) due to an imbalance between prooxidants and antioxidants in vivo causes oxidative modification of lipids, proteins, DNA, etc., leading to accelerated cellular death. Oxidative stress (OS) promotes the development and progression of excitotoxicity, leading to oxidative damage to lipids, proteins and DNA, disruption of the blood-brain barrier, and the initiation of secondary mechanisms including neuroinflammation, apoptosis, and ferroptosis, as well as autophagy dysfunction. Formed at inflammatory sites, oxidants such as superoxide (O_2_^•−^) and hydroxyl (^•^OH) radical, hydrogen peroxide (H_2_O_2_), and hypochlorous acid (HOCl) appear to contribute to the tissue damage in some acute and chronic inflammatory diseases [1].

The primary defense comprises three key enzymes that prevent free radical formation or neutralize them: glutathione peroxidase (GPx), catalase (CAT), and superoxide dismutase (SOD). GPx donates two electrons to reduce peroxides by forming selenols and eliminates peroxides that could otherwise act as substrates for the Fenton reaction. CAT converts H_2_O_2_ into H_2_O and O_2_. Lastly, SOD converts superoxide radical into H_2_O_2_, which then serves as a substrate for CAT [2].

The brain is particularly sensitive to oxidative damage, due to its high levels of polyunsaturated fatty acids, which represent one of the prime targets for ROS [3]. OS is a key factor that must be addressed after ischemic brain injury. Numerous preclinical studies have demonstrated that OS following the accumulation of ROS during ischemic stroke (IS) irreversibly damages cellular biomolecules, including lipids, proteins and DNA, leading to neuronal death. In the early stages of IS, increased oxygen supply to ischemic foci leads to a rapid increase in oxygen radicals, which cause DNA damage and trigger a cellular mechanism known as the DNA damage response. This mechanism involves homologous recombination and non-homologous end joining to efficiently repair damage.

Additionally, poly(ADP-ribose) polymerase 1 (PARP-1), a key member of the poly(ADP-ribose) polymerase superfamily, plays a critical role in both DNA repair pathways and the regulation of cellular apoptosis. Elevated PARP-1 levels following apoptosis induction trigger the translocation of apoptosis-inducing factor (AIF) to the nucleus. This leads to chromatin disruption and cell death independent of caspase activation via binding to the histone H2A variant H2AX. The PARP/AIF pathway mediates cell death independently of caspases, a process known as parthanatos. Neuronal DNA damage caused by OS during ischemia is closely linked to parthanatos [3,4].

Lipid peroxidation (LPO) can be described as a process in which oxidants (primarily ROS) attack lipids containing carbon–carbon double bonds. Therefore, various antioxidant substances have been shown to reduce ischemic brain damage. The antioxidant defense system neutralizes or scavenges ROS and plays a protective role against ischemic brain damage. One of the key enzymes in this system is SOD, which primarily functions to protect against the harmful effects of OS. Altering SOD expression has demonstrated significant success in animal models of transient cerebral ischemia. Sugawara et al. [5] showed that transgenic rats overexpressing SOD1 had reduced infrared-induced superoxide production and selective death of CA1 pyramidal cells. Keller et al. [6] also found that transgenic mice overexpressing SOD2 had significantly reduced LPO, protein nitration and cerebral infarction after transient focal cerebral ischemia. Increased expression of SOD1 and SOD2 in CA1 pyramidal cells after pre-treatment with natural antioxidants of plant origin (quercetin, chlorogenic acid, etc.) contributed to the cell protection from ischemic damage in gerbils.

Antioxidant therapy for acute ischemic stroke can slow the progression of Alzheimer’s-type dementia by suppressing OS, pathological tau phosphorylation and β-amyloid accumulation. Early administration of antioxidants activates Nrf2, reduces NADPH oxidase activity and neuroinflammation, maintains synaptic plasticity and the integrity of the blood-brain barrier. Clinical data show a delay in cognitive decline, especially in patients with premorbid signs of neurodegeneration [3,7].

All these factors make the development of new neuroprotective strategies crucial, both to protect brain cells from ischemia and to extend the time window for thrombolytic therapy. As a result, the Stroke Academic Industry Roundtable (STAIR) guidelines now pay special attention to agents with multiple mechanisms of action for neuroprotection. Of particular interest are endogenous bioamines—derivatives of thyroid hormones known as thyronamines—which can exert a hypothermic effect on the brain, modulate behavioral and cognitive functions and influence OS [8].

We have assumed that thyronamines can also contribute to the nervous tissue antioxidant protection. Their AO properties can be estimated using a complex approach that includes the experimental determination of AOA markers and computational methods for characterizing their structure and reactivity with ROS.

In terms of oxidative stress intensity assessment, indicators such as malondialdehyde (MDA), an OS biomarker, and endogenous antioxidants superoxide dismutase and glutathione peroxidase, which catalyzes the oxidation of reduced glutathione, are of special interest [9].

Methods and approaches of computational chemistry are actively used for the description and properties prediction of bioactive compounds [10]. Structural descriptors of different levels are proposed and utilized for AO compounds characterization [10]. Molecular structure descriptors such as bond lengths, bond angles, and torsion angles are used to describe stable conformations as well as changes in molecular geometry during chemical reactions. Some of them may differ significantly for origin molecules, complexes of reagents and transition states. Character and degree of changes can serve as markers of intra- and intermolecular interactions. Atomic charges are quantum-chemical descriptors that characterize the electrostatic component of chemical interactions. The polarity of a molecule is described by its dipole moment (μ). The energies of the highest occupied (*E*_HOMO_) and lowest unoccupied (*E*_LUMO_) molecular orbitals are descriptors used in calculations of electronic structures of molecules. In some cases, the *E*_HOMO_ values directly correlate with the ionization energy (*IE*) and characterize the susceptibility of the molecule to electrophilic attack. The *E*_LUMO_ values correlate with the electron affinity (*EA*) and characterize the susceptibility of the molecule to nucleophilic attack. *IE* and *EA* values, as well as parameters based on them, are often referred to as global reactivity descriptors [11] and used for property prediction.

Such markers of intermolecular interactions, like chemical shifts or vibrational frequencies of donor\acceptor groups, can be estimated from NMR or vibrational spectroscopy experiments or from DFT calculations. Quantitative description of the intermolecular interactions can be accomplished using the Gibbs free energy difference (Δ*G*) [10]. A more detailed quantitative description of intermolecular interactions is provided by using the enthalpy (Δ*H*) and entropy (Δ*S*) differences, which are related to Δ*G* as follows: Δ*G* = Δ*H* − *T*Δ*S*, where *T*—is the reaction temperature. The Δ*G*, Δ*H*, and Δ*S* values for a reaction or interaction can be regarded as descriptors of intermolecular interactions and can be estimated from experimental studies or from molecular modelling results.

**Figure 1:**
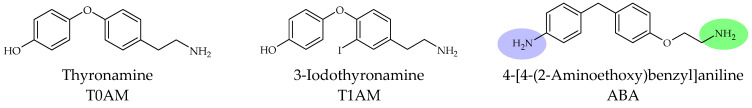
Chemical structures of biogenic thyronamines T0AM and T1AM, as well as their synthetic structural analog ABA (Figure S1). The NH_2_ group in the ABA molecule bonded with the alkyl radical is marked in green, while the one bonded with the aryl moiety is marked in violet.

The present study is a part of a systematic complex investigation focused on the structure and properties of new perspective analogs of endogenous thyronamines by experimental and computational methods. In contrast to the endogenous T0AM and T1AM thyronamines, the chosen structural analogue—4-[4-(2-aminoethoxy)benzyl]aniline (ABA) does not contain an oxygen bridge between the aryl fragments, and the 4′-OH group in it is replaced by the 4′-NH_2_ group (Figure 1). The latter has the necessary acceptor properties during hydrogen bond formation and improves the molecule’s pharmacokinetic properties (in the form of hydrochloride, hydrophilicity increases).

This paper presents the results of experimental investigations of 4-[4-(2-aminoethoxy)benzyl]aniline properties, in conjunction with molecular modeling of its geometry, electronic structure and reactivity by DFT methods. In this study, we consider the following frameworks of the problem:

– Antioxidant activity of the ABA during brain ischemia by determination of LPO markers in brain tissue and the level of neurological deficit.
– Appropriate models of ABA for the investigation of its molecular structure features and properties, as well as structural descriptors by DFT methods.

The present study is aimed:

– To evaluate the effect of the synthetic thyronamine analogue ABA on the severity of acute cerebral ischemia and its potential involvement in the regulation of antioxidant defense systems.
– To rationalize the structural models ABA for computations by DFT methods;
– To calculate descriptors of different levels for further ABA investigations.

## 2. Materials and Methods

### 2.1. Synthesis of 4-[4-(2-Aminoethoxy)benzyl]aniline

4-[4-(2-Aminoethoxy)benzyl]aniline was obtained as reported in [12]. All reagents were from Sigma-Aldrich compounds for synthesis and used without additional purification. Details on the synthesis of 4-[4-(2-aminoethoxy)benzyl]aniline and its characterization by NMR ^1^H and ^13^C spectroscopy are listed in [13].

Data on the ABA sample characterization can be found in the Supplementary Materials.

### 2.2. Bioactivity of 4-[4-(2-Aminoethoxy)benzyl]aniline

#### 2.2.1. Experimental Animals

The bioactivity of 4-[4-(2-aminoethoxy)benzyl]aniline was studied in two experiments involving a total of 58 laboratory animals (*Rattus norvegicus*). All animals were clinically healthy, with no signs of disease, and had not undergone any previous experimental procedures prior to inclusion in the study. The experimental unit was a single animal.

The animals were kept under standard vivarium conditions (air temperature 18–26 °C, relative humidity 46–65%, a 12-h daylight period) with free access to food and water, in standard plastic cages, no more than 5 animals in each.

Due to the cerebral vasculature and physiology similarities between rats and humans, and the ease of operation in animal experiments, rats were chosen as a model organism for the formation of acute ischemic stroke. Animals were randomly allocated to groups using a random number–based method. Apart from randomization, no additional measures were taken to control potential confounders.

One investigator performed the surgical procedures and was aware of group allocation. Behavioral testing and outcome assessment were conducted by two investigators blinded to group assignment. Data analysis was performed using coded data, and group identities were disclosed only after completion of the analysis.

No a priori inclusion or exclusion criteria were defined for animals. All animals that underwent the surgical procedure survived until the end of the experiment and were included in the final analyses.

All surgical and experimental procedures were performed in a dedicated laboratory room under controlled environmental conditions. No expected or unexpected adverse events were observed during the study. No predefined humane endpoints were established. Animals were monitored daily for general condition and survival.

#### 2.2.2. Oxidative Stress Assessment in the Acute Cerebral Ischemia Model

A total of 40 adult male and female Wistar rats weighing 190–210 g were used in the study. The animals were divided into two groups: control (Control) and experimental (ABA). Each group included 20 animals to account for higher biological and technical variability inherent to biochemical measurements and to ensure sufficient power to detect biologically meaningful differences. Sample sizes were determined based on experimental endpoints and prior experience.

A model of cerebral ischemia was induced by permanent ligation of the right common carotid artery (RCCA) with a silk suture. This model was selected to reproduce focal cerebral ischemia. Surgical procedures were performed under general anesthesia induced by ketamine (Calypsol) administered intraperitoneally at a dose of 80 mg/kg. The control group received a single intraperitoneal injection of 1 mL of vehicle (0.5 mL DMSO solution + 0.5 mL 0.9% NaCl solution) 10 min after ligation, and the ABA group received a single ABA solution at a dosage of 75 mg/kg, dissolved in 1 mL of vehicle. The dose was chosen based on previous studies demonstrating the thermoregulatory effect of the substance. No additional interventions were used to alleviate pain, suffering, or distress.

Twenty-four hours after the experiment, the animals were decapitated, and their brains were removed. This time period was selected to assess early oxidative stress responses following ischemic injury. The cerebral cortex tissue, separately intact and ischemic hemispheres, was homogenized and used to determine lipid peroxidation indices, specifically the content of TBA-reactive products (TBARS) and the activities of the enzymes superoxide dismutase and glutathione peroxidase.

#### 2.2.3. Assessment of Neurological Deficits

A total of 18 adult outbred male white rats weighing 200–250 g were divided into three groups: Sham, Control and ABA, 6 animals in each group. A smaller group size was employed, as behavioral outcomes exhibited lower within-group variability. This sample size was considered sufficient to detect the expected effects while minimizing animal use.

Acute cerebral ischemia was induced as described in the previous section.

Animals in the ABA group were intraperitoneally injected with ABA solution at a dosage of 75 mg/kg, dissolved in 1 mL of vehicle (0.5 mL of DMSO and 0.5 mL of 0.9% sodium chloride solution), 10 min after arterial ligation. Animals in the Control group were intraperitoneally injected with 1 mL of vehicle, 10 min after RCCA ligation.

Animals in the Sham group underwent the same surgical procedures, including the insertion of a silk thread under the RCCA. However, the artery was not ligated. Ten minutes after the ends of the silk thread were carefully trimmed, 1 mL of vehicle without ABA was injected intraperitoneally.

Modified Neurological Severity Scores (mNSS) were used to assess neurological deficits. The mNSS includes a composite of motor (muscle status and abnormal movement), sensory (visual, tactile, and proprioceptive), reflex, and balance tests. In healthy intact animals, the test score is 0. The higher the score, the more severe the neurological deficit [14]. Measurements were taken before surgery, on days 1, 3 and 7 after surgery.

#### 2.2.4. Laboratory Tests

TBA-active products were determined by reaction with thiobarbituric acid (TBA). SOD activity was determined spectrophotometrically by the ability to inhibit the reaction of adrenaline autoxidation in adrenochrome at pH = 10.2. GPO activity was assessed by changes in the amount of reduced glutathione (GSH) before and after incubation with a model substrate (*tert*-butyl hydroperoxide) using a reaction with Ellman’s reagent (5,5′-dithiobis-2-nitrobenzoic acid or DTNB). All reagents were Sigma-Aldrich compounds.

#### 2.2.5. Statistical Analysis

Statistical processing of the results was performed using the R programming language (version 4.5.2). Data distribution was assessed for normality using the Shapiro–Wilk test. As the data did not meet the assumptions of normal distribution, non-parametric statistical methods were applied. The Wilcoxon rank sum test was used to determine differences between two independent samples. The Kruskal–Wallis test was used to compare several independent samples. Dunn’s test was used for post-hoc analysis.

### 2.3. DFT Calculations

Density functional theory (DFT) calculations were carried out using the Orca software package [15]. All calculations were performed with classical B3LYP functional [16,17,18], which is still one of the best hybrid functionals [19,20] in combination with the standard Pople basis set 6-31G(d,p). Solvation by DMSO was accounted for using both the implicit model (PCM) and supermolecule approximation.

The initial structure of the ABA molecule was taken from our previous study [13] and used for ABA-H^+^ and DMSO…ABA-H^+^ models are obtained. The molecular geometry of all species was fully optimized. The absence of imaginary vibrational wavenumbers indicated that a local minimum had been reached.

Equilibrium geometries of ABA, ABA-H^+^ and DMSO…ABA-H^+^ species were used as input for the magnetic shielding constants (χ, ppm) calculations by the gauge-independent atomic orbital (GIAO) method [21]. Chemical shifts (δ, ppm) of the ^1^H for all considered species were determined on a δ-scale relative to tetramethylsilane (TMS). Full optimization of molecular geometry and calculation of χ for TMS were performed using the same level of theory and basis set (B3LYP/6-31G(d,p)/PCM).

### 2.4. Descriptors Calculations

Various structural descriptors were derived from the discrete DFT computational results. They represent the ABA properties at the atomic and molecular level (energy, bond lengths, bond and torsion angles, atomic charges, dipole moment, vibrational levels, etc.).

Global reactivity descriptors (GRDs), such as ionization energy, electron affinity, hardness (η), and softness (*S*), were calculated according to conceptual DFT using the following formulas (1)–(4). Ionzation energies refer to the vertical ionization energy, in which case the ion-radical species is in the same geometry as the neutral one, and to the adiabatic ionization energy, in which case the ion-radical species is in its lowest energy, relaxed geometry [22].

*IE* = *E*_(N − 1)_ − *E*_(N)_, (1)

*EA* = *E*_(N)_ − *E*_(N+1)_, (2)

η = (*IE* − *EA*)/2, (3)

*S* = 1/2η, (4)

where *E*_(N)_—energy of initial species, *E*_(N−1)_—energy of anion radical species, *E*_(N+1)_—energy of cation radical species.

#### Intermolecular Interactions Descriptors

Binding energies *E*_bind_ for ABA complexes with H_2_O_2_ were calculated using Equations (5) and (7), whereas the change in Gibbs Free Energy *(*Δ*G*_bind_) was calculated using Equations (6) and (8).

*E*_bind_ = *E*_complex_ − (*E*_HOOH_ + *E*_ABA…H+_), (5)

Δ*G*_bind_ = *G*_complex_ − (*G*_HOOH_ + *G*_ABA…H+_), (6)

*E*_bind_ = *E*_complex_ − (*E*_HOOH_ + *E*_ABA…H+_ + *E*_Cl−_). (7)

Δ*G*_bind_ = *G*_complex_ − (*G*_HOOH_ + *G*_ABA…H+_ + *G*_Cl−_), (8)

where *E*_complex_, *E*_HOOH_, *E*_ABA…H+_, and *E*_Cl−_—are energies of a complex, free hydrogen peroxide, ABA...H^+^ cation, and Cl^−^ anion, respectively (with ZPC); *G*_complex_, *G*_HOOH_, *G*_ABA…H+_, and *G*_Cl−_—are Gibbs free energies of a complex, free hydrogen peroxide, ABA...H^+^ cation, and Cl^−^ anion, respectively. All parameters used for calculation were obtained for species in equilibrium geometry at the B3LYP/6-31G(d,p) with D3BJ dispersion correction [23,24].

The peroxide bond dissociation energies (*BDEs*) for H_2_O_2_ and ABA complexes with H_2_O_2_ were calculated as the energy difference between the hydroxyl radicals and the H_2_O_2_ molecule as defined in Equation (9).

*BDE*_O-O_ = 2*E*_HO_ − *E*_HOOH_, (9)

where *E*_HO_—is the energy of hydroxyl radical (with ZPC), *E*_HOOH_—is the energy of free hydrogen peroxide with relaxed geometry or energy of the hydrogen peroxide conformation in the complex (with ZPC). All parameters used for calculation were obtained for optimized species at the B3LYP/6-31G(d,p) with D3BJ dispersion correction [23,24].

## 3. Results and Discussion

### 3.1. Markers of the ABA Antioxidant Activity in the Model of Acute Cerebral Ischemia

**Figure 2:**
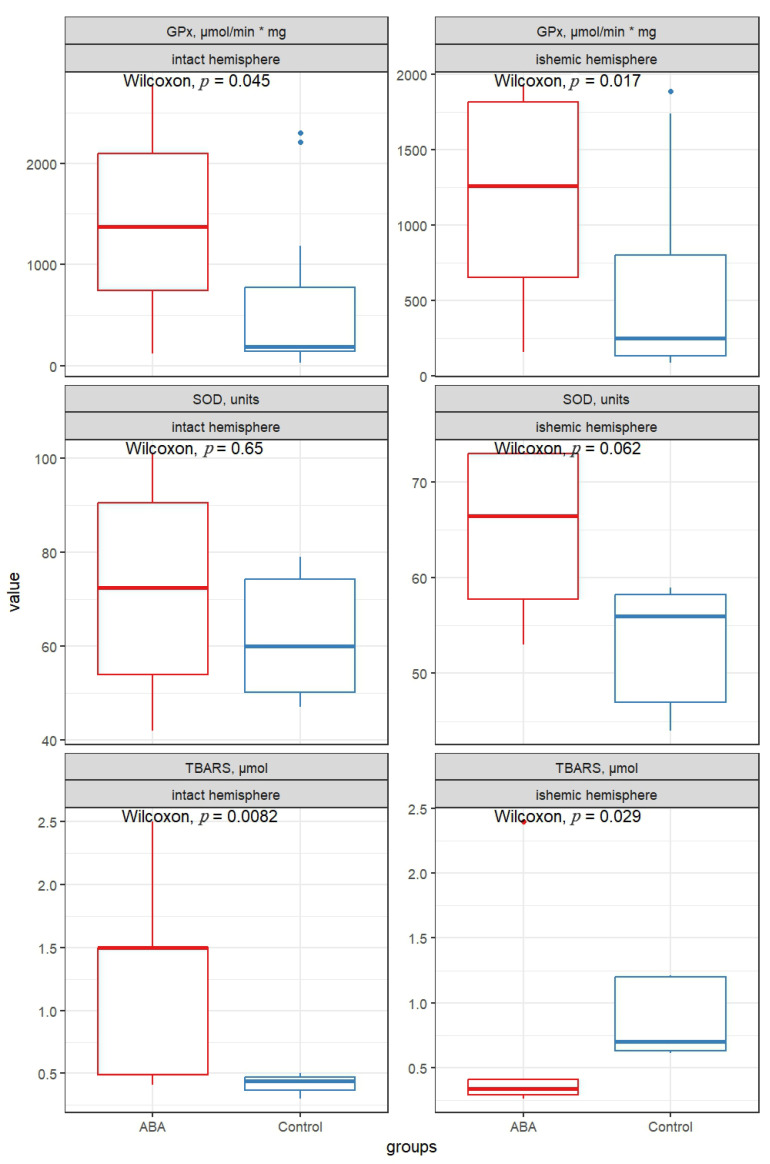
Boxplots showing antioxidant activity in the intact and ischemic hemispheres in control and ABA treatment. SOD—superoxide dismutase, GPx—glutathione peroxidase, TBARS—thiobarbituric acid reactive substances, µmol/min * mg—specific activity of the enzyme (the amount of enzyme in mg capable of converting 1 µmol of substrate in 1 min under standard conditions).

As expected, in the control group, the median TBARS level was higher in the ischemic hemisphere than in the intact hemisphere, indicating enhanced lipid peroxidation following ischemia. Administration of ABA resulted in more than a two-fold decrease in the median level of TBARS in the ischemic hemisphere compared with control animals (*p* = 0.029). In contrast, an opposite trend was observed in the intact hemisphere: ABA-treated rats demonstrated an approximately 3.5-fold increase in the median TBARS level compared with the untreated control group (*p* = 0.008). The results are presented in Figure 2 and Table 1.

No statistically significant differences in SOD activity were found in the cortex of the intact cerebral hemispheres between the control and experimental groups. However, in the ischemic hemisphere of ABA-treated animals, SOD activity was higher than in the control group, although this difference did not reach statistical significance (*p* = 0.062).

Comparison of GPx activity between the control and experimental groups indicated an increase in median values following ABA administration. In the intact hemisphere, GPx activity was elevated by more than sevenfold (*p* = 0.045), while in the ischemic hemisphere, an increase of more than fivefold was observed (*p* = 0.017).

Glutathione peroxidase plays a key role in the reduction of lipid hydroperoxides and hydrogen peroxide; therefore, its activation may contribute to limiting oxidative damage during the early phase of ischemic injury. The parallel increase in GPx activity observed in both hemispheres suggests that ABA may exert a systemic effect on the regulation of antioxidant enzymes. Although changes in superoxide dismutase activity did not reach statistical significance, the tendency toward increased SOD activity in the ischemic hemisphere of ABA-treated animals is consistent with an overall shift toward enhanced antioxidant capacity.

The decrease in TBARS levels in the ischemic hemisphere following ABA administration is consistent with attenuation of ischemia-induced lipid peroxidation. Paradoxically, in the intact hemisphere, ABA administration led to an increase in TBARS, despite elevated GPx activity and the same SOD levels. This may indicate that the effect of ABA depends on the tissue’s state (normal vs. ischemic), possibly due to its differential behavior under varying pH conditions. This observation requires further study, including the addition of intact groups of animals after drug administration, which is a limitation of our study.

### 3.2. Effect of ABA Administration on the Indicators of Neurological Deficit in an Experimental Model of Acute Cerebral Ischemia

**Figure 3:**
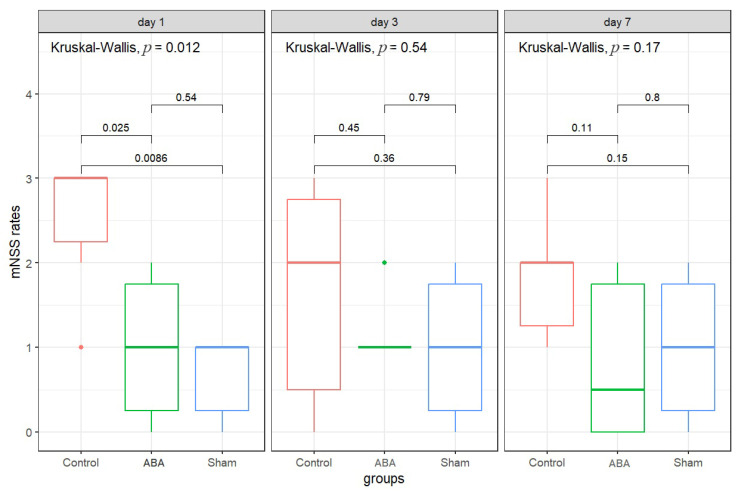
Results of the mNSS test on days 1, 3 and 7 after acute cerebral ischemia in control and ABA treatment.

The results of the mNSS test are presented in Figure 3 and Table 2.

Before surgery, the animals in all study groups showed no differences in neurological scores, with medians of less than one point. On the first day after surgery, the control group exhibited a significant increase in median mNSS score to 3 points. In contrast, the median scores in the ABA and Sham groups were both 1.0 (*p* = 0.025 and *p* = 0.009, respectively, compared to the control group). The scores of the ABA-treated animals were not statistically significantly different from those of the sham-operated group.

On the third and seventh days, the scores in the control group remained higher (1.67 and 1.83, respectively), but no statistically significant differences were found between the groups.

Thus, it can be assumed that ABA administration was associated with a reduction in neurological deficit compared to untreated controls. Reduction of lipid peroxidation and activation of antioxidant enzymes provide a possible biochemical basis for the improved neurological outcomes observed in the behavioral experiment. However, this part of the study has several limitations, including the relatively small sample size and the use of different rat strains across experiments, which may limit the direct comparability of biochemical and behavioral outcomes.

The observed effects of ABA on the regulation of redox balance during ischemic brain injury may be relevant to human pathophysiology; however, further studies are required to confirm their translational applicability.

### 3.3. Chemical Species Distribution for the ABA at Different pH Levels

**Figure 4:**
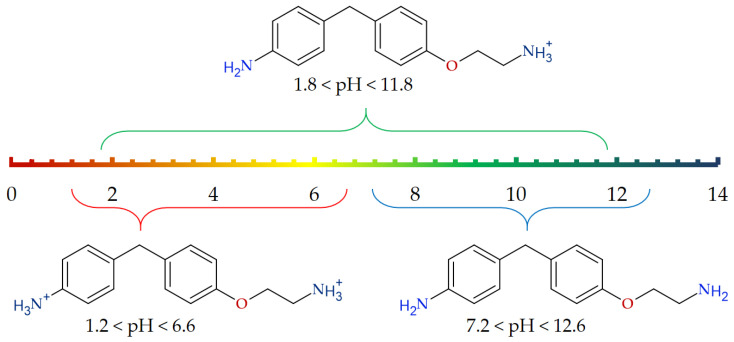
The chemical species distribution for ABA at different pH levels.

There are two NH_2_ groups (aryl- and alkyl-bonded) in the structure of ABA (Figure 1, marked in violet and green), which are sensitive to the medium pH level. Thus, one can expect the presence of several protonated forms of ABA in water solutions as well as in vivo at different pH levels. The neutral and protonated forms distribution for the ABA (Figure 4) at different pH values was estimated with the Marvin Protonation Plugin [25]. Corresponding pKa values estimated for the two considered NH_2_ groups are pKa_aryl_ = 4.39 and pKa_alkyl_ = 9.28 for the aryl and alkyl moieties. correspondingly.

Intracellular pH in the brain is maintained at approximately 7.2 [26,27,28,29]. This parameter is strongly regulated by active (ion pump transport) and passive (ion channel transport, intracellular buffer solution) mechanisms [27]. During a stroke, the extrusion of CO_2_ from the cell is limited by poor perfusion. Accumulation of CO_2_ in the intracellular space decreases the performance of the buffer solution and contributes to the reduction of pH [27]. Overall, intracellular pH in the brain is dependent upon local perfusion, intracellular energy reserves and time. A pH threshold of 6.3–6.4 exists, beyond which cellular pH-related damage is triggered [27,30]. As ABA is a promising neuroprotector for ischemic stroke treatment, it is important to estimate its possible forms under physiological conditions mentioned above. Our estimations have shown mono protonated form of ABA (ABA-H^+^) to be the main species (level up to 99%) at physiological pH 6.9–7.2 range. Thus, we consider the mono-protonated form of ABA-H^+^ as the main object for the next studies. Some parameters for the un-ionized ABA form will also be presented for comparison.

### 3.4. Structural Models of the ABA for DFT Calculations

**Figure 5:**
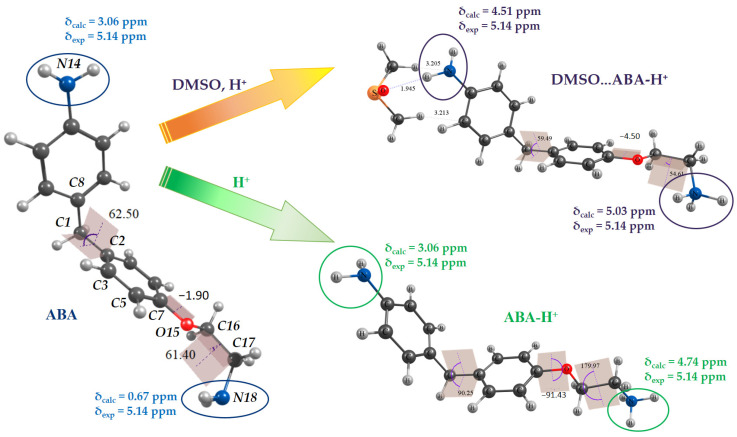
Calculated lowest-energy geometries of the ABA in neutral and protonated states, as well as a supermolecular solvation model of protonated ABA with one molecule of DMSO added explicitly into computational space at the stages of geometry optimization and calculation of ^1^H NMR chemical shifts of ABA. All calculations are carried out at the B3LYP/6-31G(d,p) level using the PCM scheme to take into account the nonspecific solvent effects of DMSO.

It was shown previously that chemical shifts of ^1^H nuclei are quite correctly reproduced for the ABA molecule at B3LYP/6-31G(d,p)/PCM level of theory [13], but with the exception of NH_2_ groups’ protons, which are sensitive to the intermolecular interactions. DMSO was used as a solvent in NMR spectroscopy studies of the ABA structure [13,31] and as a component of the vehicle for the ABA injection preparations in the present study and in our previous work [31]. Additional specific solvation of ABA by DMSO molecules considered within supermolecule approximation [31] revealed that the value of δ(NH_2_) = 5.14 ppm experimentally observed for the aniline fragment is due to specific solvation. This is why one can consider ABA, ABA-H^+^, and its solvate DMSO…ABA-H^+^ as models for DFT calculations. Equilibrium geometries of the ABA, ABA-H^+^ and DMSO…ABA-H^+^ were obtained (Figure 5) and used for the magnetic shielding constants of the ^1^H nuclei calculations. Chemical shifts of the NH_2_ groups’ protons were estimated. Good agreement between experimental and calculated δ(NH_2_) values was obtained in the case of DMSO…ABA-H^+^ (Figure 5). Thus, the equilibrium geometries considered for the ABA, ABA-H^+^ and DMSO…ABA-H^+^ species were used for further descriptor assessments.

### 3.5. Descriptors for the ABA

Molecular geometry descriptors include bond lengths, bond angles, and torsion angles, and can be obtained from geometry optimization. Torsion angles (C^8^–C^1^–C^2^–C^3^ (α), C^5^–C^7^–O^15^–C^16^ (β), and O^15^–C^16^–C^17^–N^18^ (γ)), which are crucial for the ABA, are listed in Table 3 for all considered models. Mutual arrangement of these angles determines the spatial configuration of the ABA in neutral, protonated or solvated form. Values of α, β, and γ for ABA-H^+^ significantly differ from the corresponding ones for the neutral ABA molecule. The configuration of the ethylamine fragment changed from synclinal (γ = 61.4°) to antiperiplanar (γ = 179.9°). The C–N bond length is elongated noticeably due to protonation.

Electronic structure descriptors considered for ABA forms are Mulliken atomic charges for NH_2_ groups and O atoms (q, e), total dipole moment (μ, D), energies of frontier molecular orbitals and an energy gap. The energy gap of frontier molecular orbitals (*E*_gap_) is the difference between *E*_LUMO_ and *E*_HOMO_ (*E*_gap_ = *E*_LUMO_ − *E*_HOMO_); the higher *E*_gap_, the more kinetically inert the molecule is [32].

Protonation and solvation lead to a decrease in the net negative charges on N atoms and a net negative charge on O atoms in the structure of ABA (Table 3). As expected, dipole moment values for protonated species are significantly higher compared to the neutral ABA molecule. *E*_gap_ value was found to slightly decrease in the row: ABA > ABA-H^+^ > DMSO…ABA-H^+^.

The global chemical reactivity descriptors describe the sensitivity of a compound’s structure to slight changes in the external potential and electrons. These descriptors are used to predict reactivity and structure stability [11]. The GCRDs commonly used to measure the electron-transfer characteristics of antioxidants were calculated for ABA forms. They are given in Table 3.

### 3.6. Descriptors of Intermolecular Interactions: H-Bonds Formation and Interaction with Reactive Oxygen Species

As for the different types of descriptors of intermolecular interactions, in this work, for the ABA molecule, we limited ourselves to considering well-known markers of hydrogen bonds, and also considered the interaction of ABA with HOOH as an example of ROS.

*H-bonds markers*. Vibrational (IR and Raman) and NMR spectroscopy are traditionally used to study intermolecular interactions in solutions and confirm H-bond formation [33,34]. In the case of ABA, the amino groups are the obvious reaction centers for intermolecular interactions. Therefore, the characteristic vibrations of these groups in IR spectra and the chemical shifts of protons in NMR spectra will be sensitive to their participation in H-bond formation [13,35]. Calculated harmonic vibrations wavenumbers corresponded to the stretchings and bendings of free and protonated amino groups in ABA, ABA-H^+^, and DMSO…ABA-H^+^ are listed in Table 3. Protonation and specific solvation result in the shifts of key frequencies towards lower wavenumbers and are crucial for the NH_2_ groups’ chemical shifts.

*Interaction with ROS.* 3-Iodothyronamine and 3-iodothyroacetic acid (TA1) are the only known endogenous thyroid-hormone-related compounds for which redox properties were studied [36]. TA1 showed both superoxide (O_2_^•−^) and hydroxyl (^•^OH) radical scavenging activities, and T1AM had only the capacity to scavenge superoxide radicals. These reactions may occur due to the presence of the hydroxyl (-OH) group in the structure of T1AM and the corresponding acid. This type of activity is similar to that of phenol compounds [37,38,39].

Reactivity of endogenic thyronamines as well as their synthetic analogs can also be related to H_2_O_2_ interaction reactions. ABA protonated form contains the –O–CH_2_–CH_2_–NH_3_^+^ fragment which is closely related with choline (HO–CH_2_–CH_2_–N(CH_3_)_3_^+^) and acetylcholine (CH_3_C(O)O–CH_2_–CH_2_–N(CH_3_)_3_^+^) structures. These compounds and their structural analogs have shown to demonstrate antiradical activity in a model of H_2_O_2_-induced toxicity [40]. Also, they can activate radical decomposition of H_2_O_2_ with (^•^OH) radical generation [41,42]. Counteranion (such as Cl^−^, Br^−^, or I^−^) can regulate the reactivity of choline, acetylcholine cations, as well as their structural analogs [42,43,44,45,46]. The first step of peroxide bond catalytic decomposition is the complex formation between the catalyst species.

**Figure 6:**
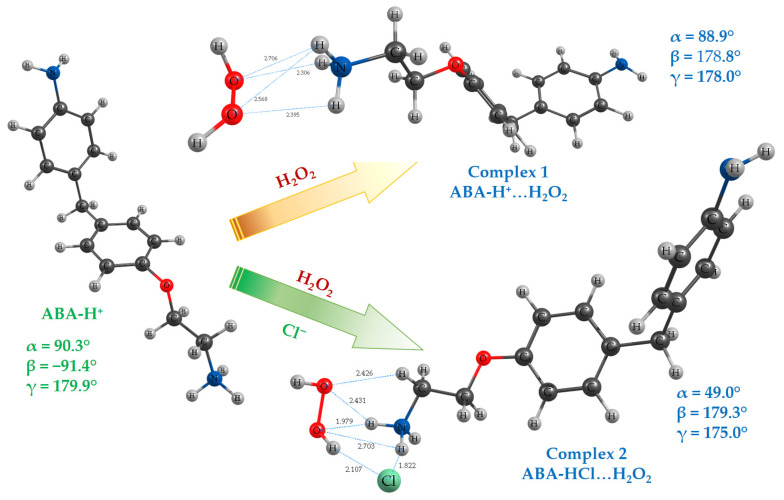
Calculated lowest-energy geometries of the ABA-H^+^ complexes with the H_2_O_2_ molecule. All calculations are carried out at the B3LYP/6-31G(d,p) level with dispersion correction.

Possible structure models of the complex between the hydroperoxide and quaternary ammonium salts were proposed based on the experimental NMR spectroscopy data [43,44,45]. In this study, we consider the interaction between the ABA protonated form and the H_2_O_2_ molecule, including ABA-H^+^…H_2_O_2_ complex formation, as well as the combined action of the two ions (Figure 6).

The complexes considered were probed for structural parameters, binding energies, and stability. The obtained results are listed in Table 4 and Table 5. Binding energies for Complexes 1 and 2, as well as structural parameters, point out multiple intermolecular interactions in the considered species. Interatomic distances between protons of the NH_3_ group and peroxide oxygen atoms are within 1.9–2.7 Å (Figure 6). Additional H-bonds are formed between Cl^−^ and the O–H group of the peroxide, as well as the NH_3_ group of protonated ABA.

It should be noted that complexation with H_2_O_2_ leads to the reorganization of the ABA structure. Key torsion angles that determine the ABA spatial configuration changed from α = 90.2° and β = −91.4° in ABA-H^+^ to α = 88.9° and β = 84.7° in the Complex 1 and α = 49.0° and β = 179.3° in the Complex 2.

Complexation with cation ABA-H^+^ or the combined action of two different centers (ABA-H^+^ and Cl^−^) on the peroxide molecule is effective for its binding without activation of O–O bond: difference ~2 kJ·mol^−1^ between *BDE*_O–O_ value for bonded and non-bonded peroxide is insignificant (Table 4). Additional experimental investigations are needed to verify these results.

According to the obtained results, the following future research directions can be highlighted:

– Experimental study of the ABA antioxidant activity in the reactions with ROS;
– Study of the ABA reactivity in the reactions with ROS by DFT methods.

## 4. Conclusions

It has been found that the T0AM thyronamine analogue, ABA, is able to control the redox status in acute cerebral ischemia, which may be a mechanism for neuroprotection of healthy tissues, contributing to the reduction of neurological deficit, but further experimental studies are needed to evaluate its neuroprotective potential. Structural models of the protonated ABA (ABA-H^+^ and DMSO…ABA-H^+^) can be used for further structural investigations by DFT methods.

The knowledge yielded by this type of work—concurrent experiments on in vivo assessment of neuroprotective profile and comprehensive DFT investigations—should help expose the molecular basis of thyronamines’ bioactivity, with a view to rationally designing new and more effective neuroprotectors for future clinical use.

## Figures and Tables

**Table 1:** Oxidative stress markers in the intact and ischemic cerebral hemispheres of rats (*n* = 20 in each group) 24 h after cerebral ischemia.

Hemisphere	Marker	Group	Median	IQR
Intact	GPx, µmol/min * mg	ABA	1373	1351
		Control	190	632
	SOD, units	ABA	72.5	36.5
		Control	60	24
	TBARS, µmol	ABA	1.5	1.01
		Control	0.44	0.1
Ishemic	GPx, µmol/min * mg	ABA	1262	1167
		Control	246	666
	SOD, units	ABA	66.5	15.2
		Control	56	11.2
	TBARS, µmol	ABA	0.34	0.12
		Control	0.7	0.57

Note: IQR—interquartile range; GPx—glutathione peroxidase; SOD—superoxide dismutase; TBARS—thiobarbituric acid–reactive substances.

**Table 2:** Neurological deficit scores (mNSS) in rats (*n* = 6 in each group) after cerebral ischemia.

Day	Group	Median	IQR
Day 1	ABA	1	1.5
	Control	3	0.75
	Sham	1	0.75
Day 3	ABA	1	0
	Control	2	2.25
	Sham	1	1.5
Day 7	ABA	0.5	1.75
	Control	2	0.75
	Sham	1	1.5

Note: IQR—interquartile range.

**Table 3:** Molecular geometry, electronic structure and global reactivity descriptors of ABA, ABA-H^+^, and DMSO…ABA-H^+^ species at the B3LYP/6-31G(d,p)/PCM level.

Parameters	ABA	ABA-H^+^	DMSO…ABA-H^+^
Global reactivity			
*IE*_adiabatic_, eV	5.05	5.12	4.84
*IE*_vertical_, eV	5.37	5.46	5.23
*EA*_adiabatic_, eV	−0.41	−0.66	−0.55
*EA*_vertical_, eV	−0.21	−0.45	−0.34
η, eV	2.73	2.89	2.70
*S*, eV	0.18	0.17	0.19
Electronic structure			
*E*_HOMO_, eV	−5.39	−5.46	−5.23
*E*_LUMO_, eV	−0.12	−0.36	−0.28
*E*_gap_, eV	5.27	5.10	4.95
μ, D	1.44	30.54	34.31
q(N^14^), e	−0.676	−0.676	−0.710
q(N^18^), e	−0.631	−0.525	−0.519
q(O^15^), e	−0.546	−0.560	−0.561
Molecular geometry			
C^13^–N^14^, Å	1.398	1.397	1.398
C^17^–N^18^, Å	1.465	1.505	1.506
N^14^–H, Å	1.012	1.012	1.011 1.022
N^18^–H, Å	1.019	1.025	1.025
α, °	62.5	90.3	59.5
β, °	−1.9	−91.4	−4.5
γ, °	61.4	179.9	54.6
Harmonic vibrations *			
sym stretching (NH_2_)_ar_, cm^−1^	3557	3557	3401
sym stretching (NH_2_)_alk_, cm^−1^	3478	3428	3417
asym stretching (NH_2_)_ar_, cm^−1^	3658	3658	3627
asym stretching (NH_2_)_alk_, cm^−1^	3558	3520 3512	3525 3502
scissoring (NH_2_)_ar_, cm^−1^	1656	1672 1654	1685
scissoring (NH_2_)_alk_, cm^−1^	1663	1674 1670	1674
wagging (NH_2_)_ar_, cm^−1^	622	627	732
stretching (N–C)_alk_, cm^−1^	1144	1015	1068
stretching (N–C)_ar_, cm^−1^	1309	1312	1324
torsions (NH_2_)_ar_, cm^−1^	302	304	-
torsions (NH_2_)_alk_, cm^−1^	325	283	271
NMR ^1^H chemical shifts			
δ (NH_2_)_ar_, ppm	3.06	3.06	4.51
δ (NH_2_)_alk_, ppm	0.67	4.74	5.03

* Notes: Calculated wavenumbers are listed without any scale factors; atom numbering corresponds to that on Figure 5.

**Table 4:** Binding energies of the hydrogen peroxide with protonated thyronamine ABA-H^+^ and strength of the peroxide bond at the B3LYP/6-31G(d,p) level.

Parameters	H_2_O_2_	Complex 1	Complex 2
*E*_bind_, kJ·mol^−1^	-	−53.8	−624.7
Δ*G*_bind_, kJ·mol^−1^	-	−8.9	−534.8
*BDE*_O-O_, kJ·mol^−1^	201.3 *	201.1	198.6

* Note: Activation energy of H_2_O_2_ radical decomposition is 201 ± 12 kJ·mol^−1^ in vapor [47,48].

**Table 5:** Parameters of the molecular structure of hydrogen peroxide and its complex with protonated thyronamine ABA-H^+^ at the B3LYP/6-31G(d,p) level with dispersion correction.

Parameters	H_2_O_2_ Experimental [49,50,51,52]	*H_2_O_2_	*Complex 1	*Complex 2
l_O-O_, Å	1.453 ± 0.007	1.456	1.457	1.455
l_O-H_, Å	0.988 ± 0.005	0.971	0.973	1.001 0.971
∠HOO, °	102.7 ± 0.3	99.8	101.0	101.3 101.2
∠HOOH, °	90.2 ± 0.6	118.6	112.0	−88.9
d (NH…O), Å	-	-	2.31 2.39	1.98 2.70
d (N…O), Å	-	-	2.81	2.79
∠ (N–H…O), °	-	-	109.0 103.2	133.9 73.6
d (OH…Cl^−^), Å	-	-	-	2.11
∠ (O–H… Cl^−^), °	-	-	-	159.6
d (NH…Cl^−^), Å	-	-	-	1.82
∠ (N–H… Cl^−^), °	-	-	-	163.7
stretching (O–O), cm^−1^	864	955	948	952
stretching (O–H), cm^−1^	3618	3763 3764	3750 3723	3766 3214
bending (O–O–H), cm^−1^	1393	1444 1314	1420 1320	1467 1366
torsion (H–O–O–H), cm^−1^	-	351	524 290	240 233
sym stretching (NH_3_), cm^−1^	-	-	3407	-
asym stretching (NH_3_), cm^−1^	-	-	3507 3466	3528 3326 2137
bending (NH_3_), cm^−1^	-	-	1660 1688	1614 1658
torsion (NH_3_), cm^−1^		-	255	460
bending (N–H…O), cm^−1^	-	-		1177
bending (O–H…Cl^−^), cm^−1^	-	-	-	904

* Note: Wavenumbers are listed without any scale factors.

## Data Availability

The original contributions presented in this study are included in the article/Supplementary Material. Further inquiries can be directed to the corresponding author(s).

## Supplementary Materials

The following supporting information can be downloaded at: https://www.mdpi.com/article/10.3390/antiox15020256/s1, Figure S1: Chemical structure of 4-[4-(2-aminoethoxy)benzyl]aniline (ABA).

## Abbreviations

The following abbreviations are used in this manuscript:

ABA	4-[4-(2-Aminoethoxy)benzyl]aniline
AIF	Apoptosis-Inducing Factor
BDE	Bond Dissociation Energy
CAT	Catalase
DMSO	Dimethyl Sulfoxide
DTNB	5,5′-Dithiobis-2-nitrobenzoic Acid
DFT	Density Functional Theory
E_bind_	Binding Energy
EA	Electron Affinity
ELISA	Enzyme-Linked Immuno Sorbent Assay
GCRDs	Global Chemical Reactivity Descriptors
GIAO	Gauge-Independent Atomic Orbital method
IE	Ionization Energy
IR	Infra Red
IS	Ischemic Stroke
LPO	Lipid Peroxidation
MDA	Malondialdehyde
mNSS	Modified Neurological Severity Scores
NADPH	Nicotinamide Adenine Dinucleotide Phosphate
NGB	Neuroglobin
NMR	Nuclear Magnetic Resonance
Nrf2	Nuclear Factor Erythroid 2-related Factor 2
OS	Oxidative Stress
PARP1	Poly(ADP-ribose) Polymerase 1
PCM	Polarizable Continuum Model
RCCA	Right Common Carotid Artery
ROS	Reactive Oxygen Species
TBA	Thiobarbituric Acid
TMS	Tetramethylsilane
TBARS	Products Actively Reacting with Thiobarbituric Acid
T0AM	Thyronamine
T1AM	3-Iodothyronamine
ZPC	Zero-point Energy Correction
