# Supplementary material for: Structural Descriptors and Antioxidant Activity Markers of 4-[4-(2-Aminoethoxy)benzyl]aniline
Source: Antioxidants (Basel). 2026 Feb 17;15(2):256. doi: 10.3390/antiox15020256 (PMC12938765; doi:10.3390/antiox15020256)
Supplement: Supplementary file 1 [file antioxidants-15-00256-s001.zip › antioxidants-4103029-supplementary.pdf]

Supplementary information

Structural descriptors and antioxidant activity markers of 4-[4-(2-aminoetoxy)benzyl]aniline

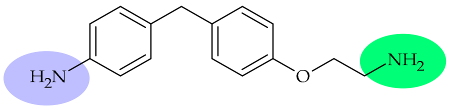

Figure S1. Chemical structure of 4-[4-(2-aminoethoxy)benzyl]aniline (ABA). NH_2 group in ABA molecule bonded with alkyl radical is marked in green while the one bonded with aryl moiety is marked in violet.

Synthesis of 4-[4-(2-aminoethoxy)benzyl]aniline

Synthesis of 4-[4-(2-aminoethoxy)benzyl]aniline was carried out in accordance with the procedure reported by Chiellini G. and co-workers [1]. All reagents were from Sigma-Aldrich compounds for synthesis and used without additional purification. Details on synthesis of 4-[4-(2-aminoethoxy)benzyl]aniline and its characterization by NMR ^{1}H and ^{13}C spectroscopy are listed in [2]. NMR ^{1}H and ^{13}C spectra of the 4-[4-(2-aminoethoxy)benzyl]aniline were recorded on a Bruker Avance instrument (400 MHz and 100 MHz) in DMSO-d_6 solution. The melting point was measured on a Stuart SMP40 melting point apparatus. Elemental analysis was performed on a Vario MICRO Cube analyzer

Yield 62% in the form of dihydrochloride, m.p. 180 °C (with decomposition). NMR ^{1}H spectrum of the base (400 MHz, DMSO-d_6), δ, ppm: 3.03 t (2H, CH_2, J 4.0 Hz), 3.69 s (2H, CH_2), 3.99 t (2H, CH_2, J 4.0 Hz), 5.14 br. s (NH_2 in exchange with water), 6.47 d (2H, H 3′,5′, J 8.0 Hz), 6.80 d (4H, H 2, 6, 2′,6′, J 8.0 Hz), 7.04 d (2H, H 3, 5, J 8.0 Hz). NMR ^{13}C spectrum of the base (100 MHz, DMSO-d_6), δ, ppm: 38.43, 39.71, 64.15, 114.05 (2C), 114.24 (2C), 128.56, 128.74 (2C), 129.20 (2C), 134.86, 146.00, 155.78. Found, %: C 57.19; H 6.37; N 8.98. $C_{15}H_{20}Cl_2N_2O$. Calculated, %: C 57.15; H 6.40; N 8.99. M 315,238.

References

1. Chiellini, G.; Nesi, G.; Digiacomo, M.; Malvasi, R.; Espinoza, S.; Sabatini, M.; Frascarelli, S.; Laurino, A.; Cichero, E.; Macchia, M.; et al. Design, Synthesis, and Evaluation of Thyronamine Analogues as Novel Potent Mouse Trace Amine Associated Receptor 1 (mTAAR1) Agonists. J. Med. Chem. 2015, 58, 5096–5107. https://doi.org/10.1021/acs.jmedchem.5b00526
2. Eresko, A.B.; Raksha, E.V.; Filimonov, D.A.; Muratov, A.V.; Voitash, A.A.; Trubnikova. N.N.; Structural Analogues of Thyronamines. Experimental and DFT-Calculated ^{1}H NMR Chemical Shifts of 4-[4-(2-Aminoethoxy)benzyl]aniline. Rus. J. Org. Chem. 2024, 60, 1654-1662. https://doi.org/10.1134/S1070428024090057
